# Propulsion and Chemotaxis in Bacteria‐Driven Microswimmers

**DOI:** 10.1002/advs.201700109

**Published:** 2017-05-24

**Authors:** Jiang Zhuang, Byung‐Wook Park, Metin Sitti

**Affiliations:** ^1^ Physical Intelligence Department Max Planck Institute for Intelligent Systems 70569 Stuttgart Germany; ^2^ Department of Mechanical Engineering Carnegie Mellon University Pittsburgh PA 15213 USA

**Keywords:** bacterial propulsion, biohybrids, collective chemotaxis, microswimmers

## Abstract

Despite the large body of experimental work recently on biohybrid microsystems, few studies have focused on theoretical modeling of such systems, which is essential to understand their underlying functioning mechanisms and hence design them optimally for a given application task. Therefore, this study focuses on developing a mathematical model to describe the 3D motion and chemotaxis of a type of widely studied biohybrid microswimmer, where spherical microbeads are driven by multiple attached bacteria. The model is developed based on the biophysical observations of the experimental system and is validated by comparing the model simulation with experimental 3D swimming trajectories and other motility characteristics, including mean squared displacement, speed, diffusivity, and turn angle. The chemotaxis modeling results of the microswimmers also agree well with the experiments, where a collective chemotactic behavior among multiple bacteria is observed. The simulation result implies that such collective chemotaxis behavior is due to a synchronized signaling pathway across the bacteria attached to the same microswimmer. Furthermore, the dependencies of the motility and chemotaxis of the microswimmers on certain system parameters, such as the chemoattractant concentration gradient, swimmer body size, and number of attached bacteria, toward an optimized design of such biohybrid system are studied. The optimized microswimmers would be used in targeted cargo, e.g., drug, imaging agent, gene, and RNA, transport and delivery inside the stagnant or low‐velocity fluids of the human body as one of their potential biomedical applications.

## 1 Introduction

Onboard micrometer‐scale actuation and powering have been remained grand challenges for miniaturization of active devices down to a few micrometer scale. However, nature has its own solutions since billions of years ago: flagellated swimming bacteria can efficiently convert chemical energy into mechanical actuation with their nanoscale biomotors. Over the past decade, numerous studies have been conducted on harnessing the flagellated bacteria, such as *E. coli* and *S. marcescens*, as propellers for biohybrid microswimmers,^1^, ^2^, ^3^, ^4^, ^5^, ^6^, ^7^, ^8^, ^9^, ^10^, ^11^, ^12^, ^13^, ^14^, ^15^, ^16^, ^17^ aiming to develop a new type of targeted drug delivery system for tumor therapy.^14^, ^16^, ^18^, ^19^ Recently, efforts have also been made to guide the motion of such bacteria‐driven microswimmers through taxis‐based^14^, ^17^, ^20^, ^21^, ^22^, ^23^, ^24^, ^25^ and magnetic steering^16^, ^26^ approaches. Among these studies, the most common way to integrate bacteria into biohybrid microswimmers is attaching intact bacterial cells onto the surfaces of synthetic microstructures, such as polystyrene microbeads, where the attachment could be enabled either by physical attraction^2^ or through chemical bonding.^14^ Current bacteria‐driven microswimmers vary vastly in materials, body shape and size, and bacterial attachment configurations; choices of these design parameters, however, have been mostly based on human intuition and empirical observation, lacking a systematic method to optimize the design of such biohybrid microsystems with respect to the their performance indicators, such as motility and guidability. To this end, it is essential to develop an analytical model that can describe the motion of bacteria‐driven microswimmers by incorporating critical design parameters, bacterial propulsion mechanics, and common guiding mechanisms such as chemotaxis.

In fluid media, free‐swimming peritrichous flagellated bacteria like *E. coli* propel themselves through a combination of running states during which the flagella form a bundle rotating counterclockwise (CCW) and propel the cell body straight forward, and tumble states, during which the flagella fall apart by clockwise (CW) rotation and the cell body is randomly reoriented. 3D tracking results of bacterial swimming motion indicate that their speed during the running states is nearly constant, but it drops significantly or almost to zero during the tumble states.^27^ Following from the linear force‐speed relation characterized by Stoke's law, we can conclude that the propulsive force of the running states is approximately constant, while the propulsive force is relatively negligible during the tumble states. Apart from the translational motion resulted from the propulsive force, the bacterial cell body also rotates due to a reaction torque, and the rotation frequency is measured to be around 20 Hz for *E. coli*.^28^, ^29^ The kinematic and dynamic quantities related to free‐swimming bacteria propulsion can be measured through visual tracking methods^28^ and optical tweezers.^29^ Although the free‐swimming propulsion of flagellated bacteria is well characterized,^30^, ^31^, ^32^, ^33^ less is known about how the attached bacteria exert forces and torques on biohybrid systems, such as bacteria‐driven microswimmers. Recently, helical‐shaped trajectories have been observed for bacteria‐driven microbeads, each with only one or a few bacteria attached,^11^ which suggests that the microbeads were propelled by near‐constant forces and torques during the helical trajectory segments. Two simple stochastic models have been proposed to simulate the swimming motion of microspheres propelled by bacteria;^34^, ^35^ however, these models fail to capture the helical motion observed experimentally, possibly due to the oversimplified constructions of such models.

Bacteria are generally sensitive to various environmental conditions, including chemoattractant/repellant, pH, oxygen level, temperature, and light. Some cues, such as spatial chemoattractant gradient, can elicit strong biased motion in bacteria, called taxis behavior, e.g., chemotaxis. Such natural sensing abilities of bacteria are considered to be ideal guiding mechanisms for the motion of biohybrid microsystems, especially for healthcare applications in the human body, where chemical cues are ubiquitous. Thus far, the taxis‐based guiding method constitutes the major way to regulate the otherwise highly stochastic motion of bacteria‐driven microswimmers, which has been studied both in vitro^17^, ^20^, ^21^, ^22^, ^23^, ^24^, ^25^ and in vivo.^14^, ^16^ Chemotaxis, one of the most common taxis behaviors in bacteria, has been well understood^36^ and its signaling pathway has been mathematically modeled.^37^, ^38^, ^39^, ^40^, ^41^, ^42^, ^43^ In general, the chemotaxis of free‐swimming bacteria associates with a biased random walk, enabled by preferentially suppressed tumble tendency when the bacteria travel up a chemoattractant gradient; whereas in an uniform medium, the tumble tendency is isotropic over all swimming directions. Chemotaxis drift has also been observed in bacteria‐driven microswimmers,^17^, ^20^, ^21^, ^22^, ^23^, ^25^ which typically consist of a spherical particle with multiple bacteria attached at random positions and orientations. Since the chemotaxis of bacteria‐driven microswimmers involves multiple bacteria whose cell bodies are interconnected through their commonly attached particle, this behavior is featured with some collective characteristics. As a result, despite the well‐established mechanisms of bacterial chemotaxis, the chemotaxis of bacteria‐driven microswimmers is not readily understood.

To shed more light on the bacterial propulsion and the chemotaxis in bacteria‐driven microswimmers, this study proposes a model to simulate the 3D motion of a multiple bacteria‐driven microswimmer system. The whole system is modeled through a combination of two subsystems: (a) multibacterial propulsion and microswimmer swimming dynamics, and (b) bacterial chemosensing and flagellar rotation dynamics. For model validation, the results of model simulations are compared to the experimental characterizations of bacteria‐driven microswimmers from different aspects of their motion behaviors: 3D swimming trajectory, motility characteristics, and chemotaxis. The simulation of the model indicates that the collective chemotaxis of the multiple bacteria attached to a microswimmer could be due to the synchronized kinase activity among these bacteria as a result of their close spatial proximity. Furthermore, we use this model to study the critical parameters that affect the performance of bacteria‐driven microswimmers, serving as the first step toward the optimized design of bacteria‐driven microswimmers for applications.

## 2 Results

### 2.1 Multicellular Propulsion Model

Most of the studies on bacteria‐driven microswimmers adopt a similar design, which is a sphere‐shaped microbead driven by single or multiple flagellated bacteria attached to it in random locations and orientations.^2^, ^6^, ^9^, ^11^, ^12^, ^13^, ^14^, ^17^, ^20^, ^21^, ^22^, ^23^, ^24^, ^25^ This particular design is chosen for its easier fabrication, characterization, and analysis, and also isotropic physical properties, such as drag coefficient, in all orientations. Therefore, we focus on modeling and experimental validation of such design of bacteria‐driven microswimmers in this study.

**Figure 1:**
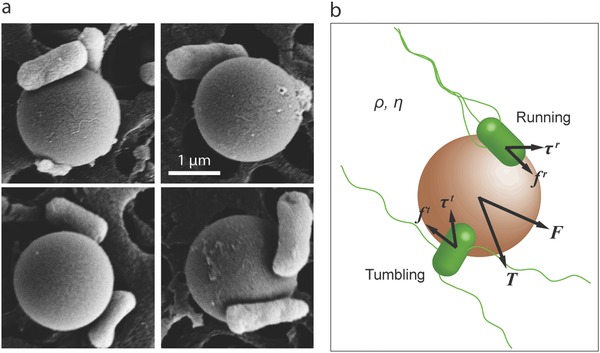
Bacteria‐driven microswimmers with a spherical body. a) SEM images showing 2 µm diameter polystyrene microbeads, each attached by a few *E. coli* bacteria. b) An illustration of the forces and torques exerted on the spherical microbead by its attached bacteria, where the force and the motor reaction torque of each bacterium are state dependent.

To simplify the system, we only consider the case that bacteria attached to the sphere are fully fixed (no position and orientation change) on the surface and perform rigid body translation and rotation with the sphere. Indeed, this condition is usually satisfied in practice by using relatively strong binding mechanisms, such as covalent (e.g., carbodiimide chemistry) and biotin‐streptavidin bindings. Scanning electron microscope (SEM) images (**Figure** **1**a) show that bacteria typically attach to spherical surfaces on their sides or with a small tilt angle, but other than that, the attachment orientation of bacteria is purely random. Flagella morphology is another important consideration in the bacterial propulsion model, because it determines the propelling forces exerted on the microswimmer. A video published by Carlsen et al.^26^ indicates a bundling behavior of flagella over the bacterial propulsion of a 6 µm diameter bead. In addition, through analysis of 3D swimming trajectories, Edwards et al. reported a near‐constant propulsive force on the microsimmers, consisting of a 5 µm diameter bead attached by a single *S. marcescens* bacterium,^11^ which suggests that the attached bacterium could be running over that period. Although the bacterial flagella could have more complicated morphologies over bacterial propulsion, based on the available observations, we conjecture that a “bundle‐and‐unbundle” dynamics, corresponding to the bacterial “run‐and‐tumble” motility, could still be the dominant flagellar morphology transition pattern. Following from this assumption, the attached bacteria can be modeled as a finite state machine with two states, running and tumbling, and the transition between these two states are determined by their chemical signaling pathway, discussed in the next subsection. The force and torques exerted by a bacterium on the sphere is state dependent, as illustrated in Figure 1b; the total propulsive force and torques on the sphere are the summed contributions of all of the attached bacteria under their current states (run or tumble). Furthermore, we adopted a relatively stiff flagella (bundle) model: each flagellar bundle has a predefined orientation but a stochastic oscillation is allowed around the predefined orientation, which tends to represent the experimental observations.^26^ However, except for introducing some white noise to simulate the stochasticity of the real system, the oscillation consideration does not affect the model behavior.

Below is a summary of the major assumptions of the bacterial multicellular propulsion model:

- Attached bacteria maintain their positions and orientations over time and perform rigid body translation and rotation together with the sphere;
- Attached bacteria transition between run and tumble states and exert different forces and torques under different states;
- Interactions between the attached bacteria on the same sphere, if exist, are small and thus negligible for studying the behavior of the microswimmers;
- Interactions between bacterial flagella and the sphere surface are negligible;
- The swimming motion occurs at low Reynolds numbers and can be approximated by Stokes' flow around a sphere, where the fluid drag due to attached bacteria is neglected;
- Physical interactions among the microswimmers are neglected due to their low concentration in medium.

With these assumptions, the instantaneous propulsive force ***F*** and torque ***T*** on a microswimmer can be described as follows

(1) $$F=\sum_{s\in \{r,t\}}\sum_{i=1}^{n^{s}}\frac{{\bar{f}}_{i}^{s}+{\tilde{f}}_{i}^{s}}{\left||{\bar{f}}_{i}^{s}+{\tilde{f}}_{i}^{s}|\right|}||{\bar{f}}_{i}^{s}||,$$

(2) $$T=\sum_{s\in \{r,t\}}\sum_{i=1}^{n^{s}}\frac{{\bar{\tau }}_{i}^{s}+{\tilde{\tau }}_{i}^{s}}{\left||{\bar{\tau }}_{i}^{s}+{\tilde{\tau }}_{i}^{s}|\right|}||{\bar{\tau }}_{i}^{s}||+r_{i}^{s}\times \frac{{\bar{f}}_{i}^{s}+{\tilde{f}}_{i}^{s}}{\left||{\bar{f}}_{i}^{s}+{\tilde{f}}_{i}^{s}|\right|}||{\bar{f}}_{i}^{s}||,$$

where *s* indicates bacterial state, either running (*r*) or tumble (*t*); *n* ^*s*^ is the number of bacteria currently in state *s*; ${\bar{f}}_{i}^{s}$ and ${\bar{\tau }}_{i}^{s}$ are the predefined force and torque of the *i*th bacteria under state *s*, respectively; ${\tilde{f}}_{i}^{s}$ and ${\tilde{\tau }}_{i}^{s}$ are the oscillation force and torque of the *i*th bacteria under state *s*, respectively; and $r_{i}$ is the position vector of the *i*th bacteria under state *s* with respect to the sphere frame. Described in Equation (1), the current force vector of a bacterium is determined by its predefined force $\bar{f}$, where the direction is aligned with the longitude of the cell body, plus a small oscillation component $\tilde{f}$, which is a random vector perpendicular to $\bar{f}$; the magnitude of the current force is regularized to an measured average value, $\left||\bar{f}|\right|$. The instantaneous resultant propulsive force ***F*** on the sphere is computed by summing up the current propelling forces of all of the attached bacteria. The force denotation and modeling rules are also applied to the motor reaction torque τ (a torque exerted by a rotating flagellum or flagellar bundle on its attaching cell body), as shown in the first term in the summation of Equation (2), where the second term in the summation computes the force‐induced toque by the propulsion force. Thus, the torque contribution of a bacterium includes two parts: the motor reaction torque and the force‐induced torque; summing them over all of the attached bacteria gives the instantaneous driving toque ***T*** on the sphere, as described by Equation (2).

Bacteria‐driven microswimmers typically operate at Reynolds numbers below 10^−4^, in which inertial effects are neglected and fluid motion is governed by the Stokes equation. Considering the spherical rigid body in our model, instantaneous fluid drag force ($F_{\mathrm{drag}}$) and torque ($T_{\mathrm{drag}}$) can be expressed in terms of the velocity and the angular velocity of the moving sphere, respectively, as follows

(3) $$F_{\mathrm{drag}}=-6\pi \eta Rv,$$

(4) $$T_{\mathrm{drag}}=-8\pi \eta R^{3}\omega ,$$

where η is the dynamic viscosity of the fluid medium, *R* indicates the radius of the sphere, and v and ω are the instantaneous velocity and the angular velocity of the sphere. Because the drag force and torque are instantaneously balanced by the propulsive force and torque, respectively, i.e., $F\;=\;-F_{\mathrm{drag}}$ and $T=-T_{\mathrm{drag}}$, we can infer the instantaneous velocity and angular velocity of the sphere from its current propulsive force and torque. At each time step of the model simulation, rigid body translation and rotation are performed for the sphere and the attached bacteria to update their positions and orientations.

### 2.2 Bacterial Chemotaxis Model

Although bacterial attachment to microbeads could block some of the ligand binding sites of some methyl‐accepting chemotaxis proteins (MCP, a transmembrane protein of bacteria for sensing extracellular concentrations of molecules and transducing the signals to intracellular regulators) physically, it is unlikely that their signaling pathway, which occurs mostly inside the cell body, can be significantly affected by the attachment. Therefore, we adopted the chemotaxis signaling pathway models established recently as the basic component of chemical sensing and response for the bacteria attached to microswimmers. But before integrating the signaling pathway models with the bacterial propulsion model to simulate the motion of bacteria‐driven microswimmers, we studied the chemotaxis response of free‐swimming bacteria via both simulations and experiments, to verify the model and to estimate the model parameters. Details of the signaling pathway models are described in the Materials and Methods section, and benchmark tests were performed to verify the normal behaviors of the model^43^, ^44^ (Figure S2, Supporting Information).

**Figure 2:**
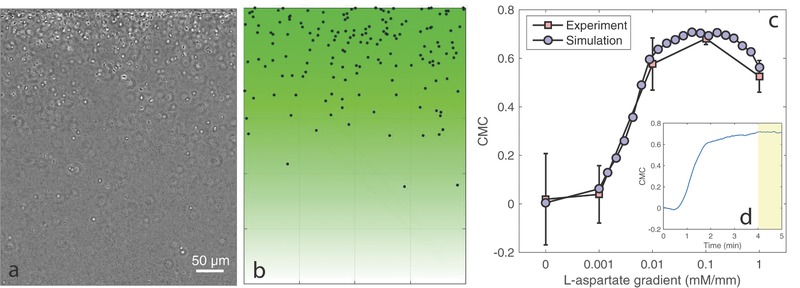
Bacterial chemotaxis in a bounded channel with a linear chemoattractant gradient. a) Steady‐state distribution of *E. coli* under a linear concentration gradient of L‐aspartate, where the white and black blobs indicate bacterial cell bodies. The linear gradient is 0.1 mM mm^−1^ and is along the vertical direction of the image, with the top having a higher concentration. b) Simulated steady‐state distribution of bacteria under the same gradient condition (i.e., *E. coli* against 0.1 mM mm^−1^ L‐aspartate), where the black dots indicate bacteria and the color profile represents the concentration gradient field. c) Bacterial chemotaxis response, measured in CMC, over different magnitudes of linear L‐aspartate gradients. The inset (d) shows a typical CMC dynamics during simulation, and the chemotaxis response (CMC) was measured from the yellow‐shaded region, where a steady‐state distribution was formed.

The chemotaxis response of free‐swimming *E. coli* bacteria was tested in a three‐channel chemical concentration gradient generator,^45^, ^46^ which has been widely used to characterize bacterial chemotaxis. The device generates a 1D stable linear gradient in a quiescent microfluidic channel, featuring a minimum yet controllable condition for observing bacterial chemotactic drift. Our previous study shows that the bacterial distribution in the test channel can reach a steady state,^47^ resulting from a balance between bacterial random diffusion and chemotactic drift. Here, we characterized the steady‐state distribution of *E. coli* against different magnitudes of linear L‐aspartate gradients, using a quantity called “chemotaxis migration coefficient (CMC),”^45^ which quantifies how the distribution is biased along the gradient. CMC is defined as CMC = ∑_*i*_(*x*_*i*_ − *x*_0_)/*w*, where *x*_*i*_ indicates the position of the *i*th bacteria along the direction of interest (i.e., the gradient direction), *x*_0_ is a reference position, usually defined to be the middle of the channel, and *w* is the width of the channel. **Figure** **2**a is an example image showing the biased distribution of bacteria under a gradient of 0.1 mM mm^−1^ along the vertical direction. For each experimental data point showed in Figure 2c, we computed the CMC based on hundreds of such distribution images, and multiple samples were analyzed to obtain the averages and the standard deviations. Side by side, the simulation results of biased bacterial distribution and chemotactic response are presented in Figure 2b,c, respectively (details about the simulation conditions are explained in the Experimental Section). The inset Figure 2d (simulation) traces the dynamic transition of the bacterial distribution before it reaches a steady state, as shaded in yellow; the CMC data in Figure 2c were calculated based on steady‐state distributions. In the simulated pathway model, the methylation and demethylation rates *K*_*R*_ = *K*_*B*_ = 0.008 s^−1^, as model parameters,^42^ were determined by creating the best fit of the simulation to the experimental data over the entire range of gradients. From the close match displayed between the model simulation and the experimental result, we can conclude that the fitted model is able to describe the chemotaxis response of the studied bacterial type toward L‐aspartate.

### 2.3 3D Motion of Bacteria‐Driven Microswimmers

**Figure 3:**
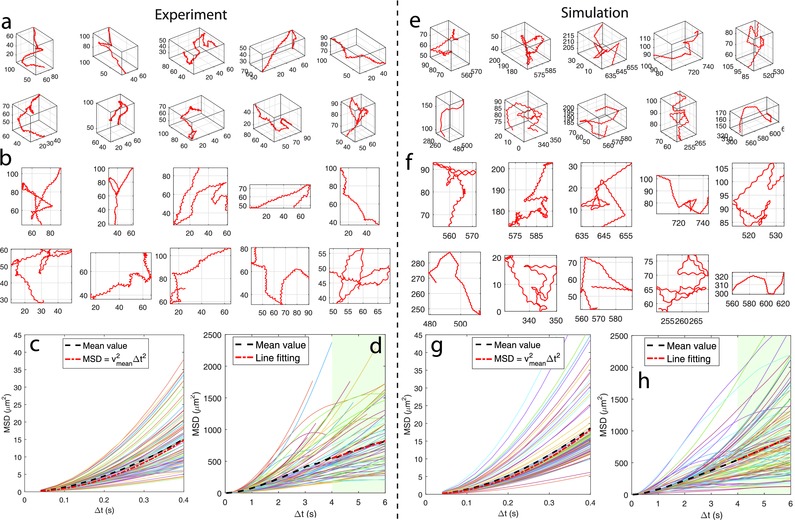
Motion of microswimmers propelled by a few (1–3) attached bacteria. a) 3D trajectories and b) their *xy*‐plane (2D) projections of 10 example microswimmers, where the length unit of each panel is µm. c) 3D mean squared displacement (MSD) plots of all of the experimentally collected tracks and swimming speed fitted in the ballistic regime. d) 3D MSD plot and random motility estimation by line fitting in the diffusive regime (green shaded). Panels (e)–(h) show the corresponding results from the model simulations, where the number of microswimmers is comparable with that of the experiments.

3D swimming trajectories of microswimmers driven by a small number of bacteria were examined to validate the proposed multicellular propulsion model. The microswimmers were fabricated by randomly attaching *E. coli* bacteria to 2.2 µm diameter Poly(methyl methacrylate) (PMMA) microbeads, through a bioconjugation using biotin–streptavidin binding; each of the microswimmers had one to three bacteria attached, observed from SEM images. The 3D motion of the microswimmers were recorded and visually tracked using a digital holographic microscopy (DHM) system. A total of 87 trajectories were collected (see Video S1 in the Supporting Information for all of the trajectories), and 10 sample trajectories are plotted in **Figure** **3**a,b, in 3D and 2D, respectively; the trajectory length varies from 4 to 40 s. As can be seen from the plots, one prominent shape of the trajectories is helical or near‐helical, similar to what has been reported by Edwards et al.^11^ on 5 µm diameter beads propelled by *S. marcescens* bacteria. A basic free body diagram of the system indicates that, while tracing helices, the propulsion dynamics of the microswimmers is dominated by a near‐constant force and a near‐constant torque which are not colinear. As another important characteristic of the trajectories, there are apparent interruptions between the consistent helices, which provide drastic reorientations for the helices; this phenomenon resembles the tumbling behavior of free‐swimming bacteria. The sudden divergence from a previous helical orientation is due to changes in the exerted force and torque, and presumably, it is the flagellar morphological transformation that causes the propulsion changes. Enlightened by this observation, our model indeed incorporates a mechanism that allows changes in the propulsive force and torques exerted by a bacterium upon its state transition. For the running state, the average propulsive force $\left||\bar{f}|\right|$ and motor reaction torque $\left||\bar{\tau }|\right|$ were estimated from the stable helical trajectories produced by single bacteria‐driven microswimmers,^11^ using the instantaneous speed and the approximated period of a helix (Figure S1, Supporting Information). In the tumble state, the average propulsive force was set to zero, considering the near‐zero translation during bacterial tumble.^27^ Although there could be a small yet to be characterized net force on tumble, our zero‐approximation is not expected to affect the model behavior. The average motor reaction torque was reduced by a factor based on the difference of flagellar rotational frequency.^28^ The average propulsive force and motor reaction torque used in our simulation are summarized in **Table** **1**. The magnitudes of the oscillation components in Equations (1) and (2) are set to be about 10% of the corresponding predefined components to mimic the system uncertainties.

**Table 1:** Average propulsive forces and torques used in the model simulation

Parameter	Symbol	Value
Average propulsive force on run	$\left||{\bar{f}}^{r}|\right|$	0.3 pN
Average motor reaction torque on run	$\left||{\bar{\tau }}^{r}|\right|$	0.7 pN µm
Average propulsive force on tumble	$\left||{\bar{f}}^{t}|\right|$	0 pN^27^
Average motor reaction torque on tumble	$\left||{\bar{\tau }}^{t}|\right|$	0.4 pN µm^28^

**Figure 4:**
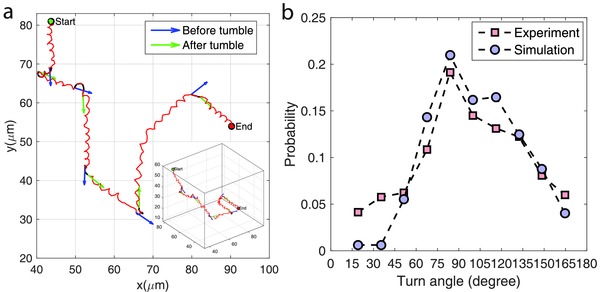
Tumble reorientation angle of bacteria‐driven microswimmers. a) A sample trajectory with five tumbles detected, and the orientations before (blue) and after (green) each tumble are marked with arrows. b) A comparison of probability distribution of tumble angles between experiment and simulation, where the most probable tumble angle lies around 80°. About 90 trajectories were assessed in both experiment and simulation.

We simulated the model with conditions similar to those of the experiments (see details in the Experimental Section), and compared the trajectory pattern/shape and the motility characteristics, including the mean squared displacement (MSD) and tumble angle, between simulations and experiments to validate the propulsion model. Figure 3e,f presents ten simulated sample trajectories in 3D and 2D, respectively, whose length is about 15 s, matching the average length of the experimentally captured trajectories. Clearly, the model reproduces the helical pattern that are generally observed along the trajectories from experiments shown in Figure 3a,b, and the simulated helices resemble the measured ones in terms of the pitch and size of helix turns. Considering the likely random reorientations occurred among the helical tracks, motility of the microswimmers was studied using MSD, the most common motion measurement for random walk systems. As shown in Figure 3, the MSD of microswimmers is examined within two different regimes, ballistic and diffusive regimes, which are determined by a characteristic time τ_*R*_ = 1.26 s, estimated through fitting the MSD formula to the simulation data.^48^ Manifested by the quadratic shape of the MSD plots over short time intervals (shorter than $\frac{1}{3}\tau_{R}$), as shown in Figure 3c,g, the microswimmers exhibit ballistic behavior, and the fitted mean speed of the simulation, *v*_mean_ = 9.9 µm s^−1^, approximates that of the experiment, 10.9 µm s^−1^. The MSD over larger time intervals (longer than 3τ_*R*_) traces a linear profile, as shown in Figure 3d,h, revealing a diffusive motility of the swimming motion over long time scales. The effective diffusion coefficients drawn from line fittings are 5.0 µm^2^ s^−1^ for simulation and 3.7 µm^2^ s^−1^ for experiment; the lower experimental value is caused by the fact that the faster microrobots are hard to track for a long time, as shown in Figure 3d, and thus biasing the long‐time data toward slower instances. Because of this bias, it is unsuitable to fit a general MSD over the full time scale of the experimental data. Besides, it has been shown that the random diffusivity of the “run‐and‐tumble” type of random walk strongly depends on the reorientation angle following a tumble event.^49^ Thus, we analyzed the helical reorientation angle of the swimming trajectories, as demonstrated with a sample trajectory in **Figure** **4**a. Figure 4b shows the probability distributions of the reorientation angle, where the simulation closely matches the experiment and both of them appear to follow a normal distribution; the most probable reorientation angle is around 80, showing a slight directional persistence between two consecutive runs.^50^ The comparable trajectory and matched motility characterizations imply that the propulsion model captures the fundamental mechanisms associated with the physical system of multi‐bacteria driven microswimmers.

### 2.4 Chemotaxis of Multicellular Microswimmers

By assuming that the individual bacteria attached to a microswimmer sense chemical concentrations in their local microenvironments and perform signal transduction independently, we propose an integrated model for the chemotaxis of multi‐bacteria driven microswimmers, which combines the multicellular propulsion model and the bacterial chemotaxis pathway model. All of the model parameters were derived from the chemotaxis of free‐swimming bacteria and bacteria‐driven microswimmers under isotropic conditions. The model was simulated and qualitatively validated through comparisons with the chemotaxis experiment data from our previous study.^25^ Specifically, we considered the situation of randomly placing a swarm of microswimmers in a bounded channel holding a linear chemoattractant concentration gradient and observing how their distribution changed over time, mainly along the direction of interest, i.e., along the gradient (see Video S2, Supporting Information) for a sample simulation).

**Figure 5:**
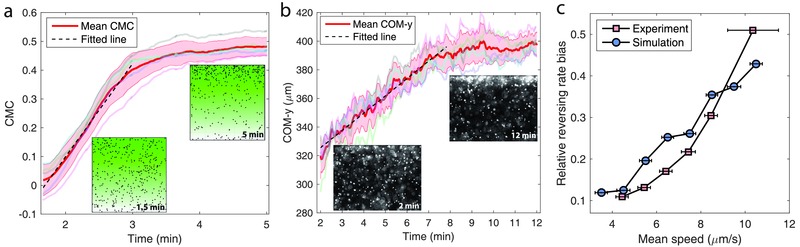
Chemotaxis of multibacteria driven microswimmers. a) The drifting dynamics (quantified in CMC) of a swarm of simulated microswimmers in a channel with a linear chemoattractant gradient. The mean CMC is assessed from five independent samples, and the red shading represents their standard deviation. The two insets show the distribution profile of the microswimmers at 1.5 min, when they are randomly located, and 5 min, when the distribution is biased toward the side with a higher chemoattractant concentration. b) Experimental measurement of the drifting process of a swarm of microswimmers propelled by *S. marcescens* bacteria. The two insets are fluorescent images representing the distribution profile of the microswimmers at 2 min (initial stage) and 12 min (final stage). This figure is reproduced with permission.^25^ Published under CC‐BY 4.0 license, copyrighted by the authors. c) Dependence of the relative direction reversing rate bias of the microswimmers on their swimming speed.

**Figure** **5**a,b show the drifting dynamics of a swarm of microswimmers from simulation and experiment, respectively, where the microswimmers are made of 3 µm diameter microbeads driven by 6–12 attached bacteria. Although the two quantities, CMC and COM‐*y* (the *y*‐component of center of mass, computed as the mean *y*‐position of all microswimmers in the field of view), are defined differently, they are all linear functions of the microswimmers' positions, and thus linearly related to each other. Both results show that, starting with a nearly uniform distribution, the swarm undergoes an approximately linear drifting process, associated with a constant chemotactic drift velocity, and eventually it settles down to a biased distribution, in which the microswimmers are more concentrated on the side with a higher concentration of the chemoattractant. Note that such a final distribution is formed because the gradient is bounded by walls, which constrain the motion of the microswimmers. The simulated drifting process, however, appears to be faster than the experimental one, reaching a steady state distribution after 4 min as compared to 8 min for experiment; this could be resulted from the fact that, compared with a simulated ideal system, the experimental system of microswimmers bears various imperfections, such as the existence of non‐motile instances and the aggregations of microswimmers. The analysis on the simulated trajectories suggests that the swimming direction of a microswimmer is more persistent when it travels up the gradient than when it moves reversely, and this is consistent with experimental observations.^24^, ^25^ When projected onto one axis, such biased motion can be quantified by the “relative reversing rate bias,”^24^ defining the bias in the direction reversing rate over the heading of an object performing an 1D random walk; the quantity contributes to the chemotactic drift velocity as a linear factor.^51^ As shown in Figure 5c, the simulation and experiment agree that the relative reversing rate bias increases with the mean speed, which in further means that the chemotactic drift velocity is enhanced on faster microswimmers.

**Figure 6:**
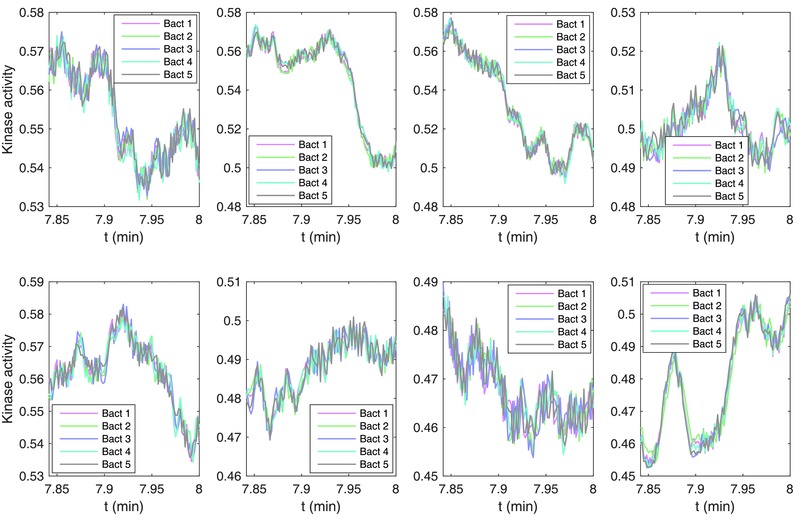
MCP kinase activity of the multiple bacteria attached to a microswimmer (simulation). Eight different microswimmers are presented, where each microswimmer has five bacteria attached randomly in both position and orientation.

Although the chemotactic drift of bacteria‐driven microswimmers can be phenomenologically explained by the heading persistence bias, it is not readily understandable why the multiple bacteria attached to a microswimmer in random orientations can perform chemotaxis in a seemingly cooperative fashion. However, our model, which successfully traces the chemotaxis of the microswimmers, rules out any explicit chemical or physical interactions among the attached bacteria on a microswimmer. But the group of physically interlocked bacteria must have an implicit avenue to “agree” with each other in order to perform chemotaxis, because the otherwise random competition among them would lead to a pure random walk in the microswimmer. Since the integrated model is supposed to explicitly account the essential biophysical components of the multibacteria driven system, all of the states of each involved bacterium is observable throughout a simulation, and thus we can identify the functioning mechanism that enables them to agree on chemotaxis. A quick conjecture is that such consensus is implicitly obtained through their mechanical bonding, by attaching to the same microbeads. To prove this, we plotted the MCP kinase activity dynamics of the bacteria attached to a microswimmer; as shown in **Figure** **6**, eight different microswimmers were examined. Interestingly, despite their independent signaling pathway, the kinase activities of the group of bacteria attached to the same microbead are highly synchronized, and such synchronization tends to produce a downstream synchronization in the tumble tendency among these bacteria. Presumably, the average tumble rate of the multiple bacteria attached to a microswimmer is higher when the microswimmer translates down the chemoattractant gradient than when it moves up the gradient, which causes a bias in the direction persistency of the microswimmers as we have observed.^25^ Based on the construction of the signaling pathway model, the pathway response synchronization must be produced by a similar concentration input trace for the group of bacteria, which is indeed guaranteed as they are interlocked in close proximity to each other by the microbead. Therefore, it is highly possible that the synchronization of signaling pathways, which explains the chemotaxis in our model, could be the reason for the chemotaxis in the experimental system, given the biophysical formulation of the model and the high level resemblance between the model simulation and experiment.

**Figure 7:**
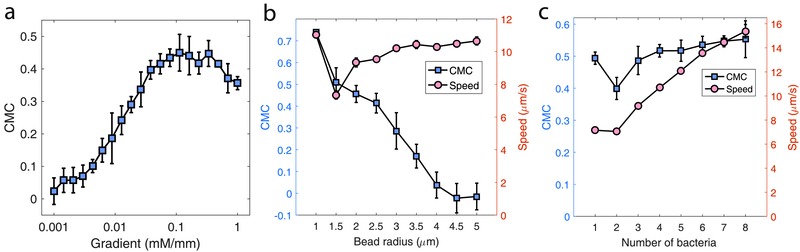
The model simulation for performance of microswimmers on system parameters. a) Chemotaxis response of microswimmers over different chemoattractant concentration profiles. Dependencies of chemotaxis and motility on b) the size of microswimmers and c) the number of bacteria attached to a microswimmer. Chemotaxis (CMC) is evaluated on the final distribution of the simulated microswimmers in the bounded channel. Unless studied as a parameter or stated otherwise, the default configuration of the simulations is 3 µm diameter microbeads, with around 5 bacteria attached to each microswimmer, and under an chemoattractant gradient of 0.1 mM mm^−1^.

The development of bacteria‐driven microswimmers has been based on experimental intuition thus far, and the dependencies of microswimmers' performance on system parameters have not been addressed, leaving the design optimization of the biohybrid system challenging. Here, we propose an approach to optimize the design of bacteria‐driven microswimmers through model‐based simulation. Chemotactic guidability and motility are the two most important considerations for the application of the bacteria‐driven microswimmers; therefore, focusing on these two performance indicators, we studied their dependencies on certain system parameters that could be easily configured in experiment. **Figure** **7**a shows the chemotaxis response of microswimmers over different chemoattractant concentration gradients, which has a profile similar to that of free‐swimming bacteria (Figure 2c), and the optimal concentration gradient for the chemotactic guidance of microswimmers is around 0.1 mM mm^−1^. However, for the microswimmers that are significantly slower than free‐swimming bacteria, we expect their strongest chemotaxis occurs at a higher concentration gradient (see Discussions). The dependencies of chemotaxis and speed on the size of microswimmers is presented in Figure 7b, where the number of bacteria attached to a microbead is proportional to its surface area and the number density is 1 bacterium per 7 µm^2^. It shows that increasing the body size of microswimmers does not affect the motility notably but causes significant decrease in CMC, almost dropping to zero for a body size over 9 µm in diameter. Larger body size would increase the average distance between cells, making the attached bacteria sense rather different concentrations and hence reducing the synchronization of the signaling pathway behavior among them (Figure S3, Supporting Information). This indicates that, to achieve better chemotactic guidance, microswimmers with smaller body size are strongly preferable. Finally, we examined the effect of the number of bacteria attached on the chemotaxis and motility. Figure 7c shows that the CMC is generally insensitive to the number of bacteria, while the speed increases almost linearly with the number of bacteria. However, the potential interactions among the attached bacteria would become more and more prominent as the number of bacteria increases, and since such interactions are not accounted in our model, the trends in Figure 7c might not hold true for any arbitrary ranges.

## 3 Discussion

We have developed a mathematical model to describe the propulsion mechanisms of a biohybrid microswimmer driven by a few attached flagellated bacteria. The simulations of the model produces 3D trajectories and motility characteristics that resemble those of experiments. The model, in combination with the signaling pathway models of bacterial chemotaxis, also traces out the chemotaxis behavior of bacteria‐driven microswimmers reported by recent studies.^14^, ^17^, ^20^, ^21^, ^22^, ^23^, ^24^, ^25^ The agreement between simulation and experiment implies that our model assumptions are reasonable and the model captures the fundamental biophysical mechanisms of the system. Furthermore, our simulation data suggests that the seemingly cooperative chemotaxis of multiple bacteria attached to a microswimmer could be explained by a synchronized signaling pathway response among these bacteria. However, proof of such prediction may need molecular level characterizations of the bacterial cells that are operated under a similar condition. In addition, the model reveals the potential dependencies of the microswimmers' performances (motility and chemotactic guidability) on system parameters, including the chemoattractant gradient, microswimmer's body size, and number of bacteria attached; such dependencies may offer useful clues for the optimized design of bacteria‐driven microswimmers.

In the multicellular propulsion model, we adopted a relatively stiff model for the bacterial flagellar bundle, which has a preferred orientation that is not affected by its local fluid field. This choice, in part illuminated by an experimental observation, appears to be rather critical for the resulting motion behaviors of the model. However, the simulation based on a purely soft flagellar bundle model (i.e., the flagellar bundle is aligned with its local flow) demonstrates a similar level of chemotaxis as the stiff flagella model. This implies that a predefined flagellar bundle orientation is not a necessity to generate chemotactic drift for the model, although it might be the case that flagellar bundles bear their own preferred orientations, and so is the preferred orientations of flagellar filaments. Besides, the consideration of flagellar oscillation in the model serves to represent the noise in the real system, and including it could introduce minor irregularities to the helical shaped trajectories, but does not fundamentally alter the average motion behavior or chemotaxis of the simulated system. The flagellar motor reaction torque, however, is essential for producing the helical shaped trajectories. This has been proven on single bacterium‐driven microswimmers: an absence of the motor reaction torque would yield circular trajectories in our simulation, instead of the helical motions observed experimentally. We assumed a run‐tumble transition for the bacteria attached to microswimmers, where the tumble is crucial for creating the propulsion asymmetry for the chemotaxis of microswimmers. If, however, a non‐tumbling mutant was considered, we don't expect any chemotaxis behavior in the current model and it should be modeled differently if the non‐tumbling strain bears different strategies for chemotaxis.

Although the motion of bacteria‐driven microswimmers is highly stochastic over long timescales, their short‐term motion could be rather deterministic, as shown by the helical‐shaped tracks in^11^ and this study. Previous models^34^, ^35^ for bacteria‐driven microswimmers fail to reproduce the recurrent helical shapes along the swimming trajectories; instead, purely stochastic motions over all timescales were simulated by these models. The main reason of their failures is oversimplified representation of biophysical components in the system. For example, both models describe each bacterium attached to a microswimmer as a single point force exerted on the microswimmer, ignoring the motor reaction torque(s) associated with the rotating flagellar bundle (or flagella). In addition, considerations of stochastic/noisy contributions could possibly smear the short‐term deterministic behaviors. In the model by Cho et al., part of the propulsion on microswimmers was contributed from the free‐swimming bacteria, by stochastically colliding with the microswimmers.^35^ Likewise, a Brownian effect was considered in the model by Arabagi et al.^34^ As our major goal is to investigate how propulsion is generated for microswimmers driven by multiple attached bacteria, other unrelated contributions were mostly ruled out in both simulations and experiments.

To adopt a relatively simple fluid mechanical model in our simulation, we approximated the swimming mechanics of bacteria‐driven microswimmers as Stokes' flow around a sphere, ignoring the shape irregularities due to attached bacteria. In our evaluated size range, the body size of microswimmers (*d* > 2.2 µm) is more dominant over the width of bacterial body (*d* < 0.8 µm), and hence making it a valid assumption to ignore the fluid drag due to attached bacteria. However, as the size of bacteria becomes comparable with that of the spherical body, this model would be less accurate to describe the system. Without complicating the swimming model too much, one way to account for the fluid drag due to the attached bacteria is to introduce an empirical size factor in the current model. This treatment is not expected to change the simulated behavior significantly but it is unable to capture the shape asymmetry caused by the attached bacteria. Finite element modeling is a potential solution to produce a more accurate representation of the system. Another major assumption we made is that the interactions between bacterial flagella and the spherical body surface are negligible. The interactions could happen through flagellar attachment to the body surface or through local fluid flows, but they are hard to characterize experimentally. Based on the observation of relatively stiff flagella, we expect such interactions are small and could not affect the system behavior considerably. This assumption is also corroborated by the close match between the experiment and simulation.

Bacteria naturally sense temporal changes of an chemoattractant concentration, by comparing the presently sensed value with that of the immediate past; this temporal sensing ability allows them to sense spatially varying signals by constantly moving around. Therefore, in a given spatial concentration gradient, the magnitude of the temporal gradient seen by a bacterium is dependent on its swimming speed, and hence the chemotactic response is speed dependent. Figure S4a (Supporting Information) shows the signaling pathway states of two bacteria with rather different swimming speeds, 2 and 20 µm s^−1^, but under a same linear chemoattractant gradient; clearly, the one with higher swimming speed manifests a significantly stronger signaling pathway response. It follows from this observation that the chemotaxis of bacteria‐driven microswimmers should also be speed dependent: the higher the swimming speed, the stronger the chemotactic response, as demonstrated in Figure 5c. From the perspective of design, within a certain range, optimizing the swimming speed of bacteria‐driven microswimmers is expected to enhance their chemotactic guidability. Potential methods to enhance the speed of bacteria‐driven microswimmers include patterning the attachment location of bacteria on microswimmers, aligning the attached bacteria, and using species with higher motility.

## 4 Experimental Section

*Bacteria Culture*: *Escherichia coli* (*E. coli*) MG1655 strain (Yale University, New Haven, USA) cultured on LB agar plates (Sigma‐Aldrich) was transferred to 5 mL LB broth (Sigma‐Aldrich, St. Louis, MO, USA) and allowed to grow at 30 °C for 4 h to its exponential growth phase. The resulted liquid culture was directly diluted with PBS (Thermo Fisher Scientific, Waltham, Massachusetts, USA) for the chemotaxis response tests of the free‐swimming bacteria. To prepare bacteria‐driven microswimmers, the resultant liquid culture was washed with phosphate‐buffered saline (PBS) before mixing with particles.

*Microswimmer Fabrication*: The bacteria‐driven microswimmers were fabricated by randomly attaching *E. coli* bacteria to 2.2 µm diameter PMMA (PolyAn, Berlin, Germany) spherical particles, where the specific and strong attachment was enabled through biotin‐streptavidin binding. The fabrication process of the microswimmers is briefly described as follows. First, 1 mL of the bacterial liquid culture was washed with PBS (centrifuged at 1500 g for 5 min) for a total of 3 cycles, with a final suspension of 1 mL. Then, an aliquot of a biotinylated anti‐*E. coli* polyclonal antibody (Abcam, Cambridge, Massachusetts, UK) was added to the bacterial suspension to reach a dilution of 1:50, followed by an 1 h incubation of the mixture on a shaker (300 rpm, 30 °C). Subsequently, the bacteria‐antibody mixture was washed with PBS for 3 cycles to remove the excess, unconjugated antibodies, and the streptavidin functionalized PMMA particles were washed with PBS (centrifuged at 5000 g for 1 min) for 3 cycles to eliminate the surfactant that came with the particles. Finally, the antibody‐conjugated bacteria were mixed with the particles at an appropriate density ratio and incubated on a shaker (600 rpm, 30 °C) for 30 min to allow for biotin‐streptavidin interactions between the bacteria and particles. To enhance motility, the assembled bacteria‐driven microswimmers were suspended in a motility medium (0.01 m KH_2_PO_4_, 0.067 m NaCl, 10^−4^ m EDTA, 0.01 m glucose, pH = 7.0) for experimental observations.

*Concentration Gradient Generator*: A three‐channel concentration gradient generator was used to characterize the chemotaxis response of free‐swimming *E. coli* bacteria. Briefly, the setup contained a sample channel lying in between two side channels, a source channel and a sink channel. Two different concentrations of the chemoattractant could be applied in the source and sink channels, respectively; as the porous material between adjacent channels allows small molecules to diffuse through, a gradient would be generated across the sample channel, wherein a bacterial sample was loaded during experiments. The detailed design, fabrication procedure, and diffusion calibration of the three‐channel microfluidic concentration gradient generator can be found in a previous work.^47^

*Imaging and Visual Tracking of Bacteria and Microswimmers*: Two optical systems, an inverted microscope (Zeiss Axio Observer A1, Oberkochen, Germany) and a digital holographic microscope (DHM T‐1000, Lyncée Tec SA, Lausanne, Switzerland), were applied to study the chemotaxis of free‐swimming bacteria and the 3D motion of bacteria‐driven microswimmers, respectively. The distribution of bacteria in the sample channel of the gradient generator was observed using a 20x (NA 0.50) objective, and phase contrast images were acquired and analyzed using an in house program developed in Matlab (R2016a, The MathWorks, Inc., Natick, MA, USA). The holograms of the bacteria‐driven microswimmers were observed using a 40x (NA 0.75) objective, which gives a 2D field of view around 165 × 165 µm^2^. The numerical reconstruction was conducted by a commercial software (Koala, Lyncée Tec) to obtain the *z*‐stacked images of the view volume, which was 110 × 110 × 110 µm^3^ with a chosen *z*‐range of 110 µm. Further, the reconstructed image stacks were analyzed by an in‐house program developed in Python 2.7 to detect the 3D positions of the bacteria‐driven microswimmers in the view volume and perform 3D motion tracking.

*Simulation Setup*: The simulated environmental conditions were 20 °C in temperature and 1 cP in viscosity, simulating the room temperature and the liquid medium used in experiments, and these conditions were common to all simulations. The fluidic boundary condition, however, was different depending on the simulation. For motion study of bacteria‐driven microswimmers, no boundaries were set, which approximated the far‐wall condition of the experimental measurements. In the chemotaxis studies of free‐swimming bacteria and bacteria‐driven microswimmers, a bounded environment was simulated to mimic the microfluidic channel in the gradient generator. Specifically, the motion of the simulated agents (bacteria or bacteria‐driven microswimmers) was constrained in a box with dimensions *x* × *y* × *z* = 500 × 500 × 250 µm^3^, where the linear chemoattractant gradient was applied along the *x* dimension. Since the experimental measurements were taken far away from the walls perpendicular to the *y*‐axis and *z*‐axis, the wall effect along these two dimensions were simply set to be reflecting the agent's (bacteria or bacteria‐driven microswimmers) motion upon hitting a wall, which avoided affecting the agents' transportation along the *x*‐axis while preserved the total number of agents in the simulated box. The wall effect along the *x*‐axis (the gradient direction) was treated differently between the free‐swimming bacteria and bacteria‐driven microswimmers, based on experimental observations. Once a free‐swimming bacterium reached a wall perpendicular to the *x*‐axis, it could be trapped into swimming along the wall for a few seconds^42^ before leaving the wall. However, the bacteria‐driven microswimmers would mostly become immobilized if they swim into the *x*‐walls,^25^ and thus this wall effect was reflected as a permanent trapping in our simulations.

A program was developed in Matlab (R2016a, The MathWorks, Inc., Natick, MA, USA) to simulate the proposed models. A simulation was conducted in two steps: initialization and iteration. In the initialization step, a given number of agents were randomly placed within the simulation environment, and their associated state variables, e.g., signaling pathway states, were initialized according to the statistics of experimental measurements. The iteration step kept updating the position and other state variables of each agent, based on its local chemoattractant concentration input, the agent's swimming dynamics, and the predefined wall effects, until the end of the given simulation duration. Necessary state traces, such as positions and pathway activities, were recorded over the iteration step for further analysis.

*Chemotaxis Signaling Pathway Model*: The chemotaxis signaling pathway of *E. coli* was modeled with three major components, and their corresponding models were adapted from recent studies.^37^, ^38^, ^39^, ^40^, ^41^, ^42^, ^43^ The first component, MCP complex, was represented with a Monod–Wyman–Changeux (MWC) model^52^ to describe the allosteric effects of receptor clusters with identical receptors. Each MCP complex switched rapidly between active (on) and inactive (off) states, determined by a free‐energy difference *F* as follows^37^, ^41^, ^42^

(5) $$F(m,\;[L])\;=\;f_{m}(m)\;+\;\ln\;\left(1+\frac{\left[L\right]}{K_{a}}\right)\;-\;\ln\;\left(1\;+\;\frac{\left[L\right]}{K_{i}}\right)$$

where *m* is the total methylation level of the receptor complex, [*L*] indicates the ligand concentration, *K*_*a*_ and *K*_*i*_ are the dissociation constants of active and inactive receptors, respectively, and *f*_*m*_(*m*) = α(*m*_0_ − *m*), with α ≈ 1.7 and *m*_0_ ≈ 1,^53^ is the methylation level‐dependent free energy difference. The suggested dissociation constants are *K*_*a*_ = 3 mM and *K*_*i*_ = 18.2 µM^54^ but may be shifted slightly in simulation to match the most sensitive ligand concentration. Hence the receptor kinase activity can be expressed as^41^, ^42^

(6) $$a\;=\;\frac{1}{1\;+\;\exp(NF(m,\;[L]))}$$

where *N* is the number of receptors in a receptor complex (*N* = 6 for *Tar* receptor and *N* = 13 for *Tsr* receptor^44^). The methylation kinetics was described by^40^

(7) $$\frac{dm}{dt}=K_{R}(1-a)-K_{B}a$$

where *K*_*R*_ and *K*_*B*_ are methylation and demethylation rates of the receptor, respectively; their values were determined by fitting the experimental data on bacterial chemotaxis response, *K*_*R*_ = *K*_*B*_ = 0.008 s^−1^. The second component modeled the signal transduction from kinase activity to the concentration of Che‐Y‐P, *Y*_*p*_, and a linear relationship was adopted in the simulation,^42^, ^43^ *Y*_*p*_ = *βa*(*t*). The third component in the signaling pathway dealt with the flagellar rotation dynamics in response to *Y*_*p*_. Enlightened by Sneddon et al.,^43^ all flagella of a bacterium were treated as a single stochastic bistable system bearing the transition rates *k*_+_ and *k*_−_ to running and tumble state, respectively, which were modeled as a function of *Y*_*p*_

(8) $$k_{\pm }\;=\;\omega_{0}\exp\;\left\{\pm \left[\frac{g_{0}}{4}\;-\;\frac{g_{1}}{2}\left(\frac{Y_{p}(t)}{Y_{p}\;+\;K_{D}}\right)\right]\right\},$$

where the parameter values were ω_0_ = 1.3 s^−1^, *g*_0_ = *g*_1_ = 40 *k*_B_ *T*, and *K*_*D*_ = 3.06 µM.^43^

## Conflict of Interest

The authors declare no conflict of interest.

## Supporting information

Supplementary

Supplementary

Supplementary
