# Supplementary material for: Propulsion and Chemotaxis in Bacteria‐Driven Microswimmers
Source: Adv Sci (Weinh). 2017 May 24;4(9):1700109. doi: 10.1002/advs.201700109 (PMC5604384; doi:10.1002/advs.201700109)
Supplement: Supplementary file 1 — Supplementary [file ADVS-4-na-s001.pdf]

Copyright WILEY-VCH Verlag GmbH & Co. KGaA, 69469 Weinheim, Germany, 2017.

ADVANCED SCIENCE
Open Access

Supporting Information

for *Adv. Sci.,* DOI: 10.1002/advs.201700109

Propulsion and Chemotaxis in Bacteria-Driven Microswimmers

*Jiang Zhuang, Byung-Wook Park, and Metin Sitti**

Supplementary Information

Propulsion and chemotaxis in bacteria-driven microswimmers

Jiang Zhuang[1,2], Byung-Wook Park[2], Metin Sitti[1, 2, *]

[1]Department of Mechanical Engineering, Carnegie Mellon University, Pittsburgh, PA 15213, USA
[2]Physical Intelligence Department, Max Planck Institute for Intelligent Systems, 70569 Stuttgart, Germany

*Corresponding author: sitti@is.mpg.de

Supplementary Figures:

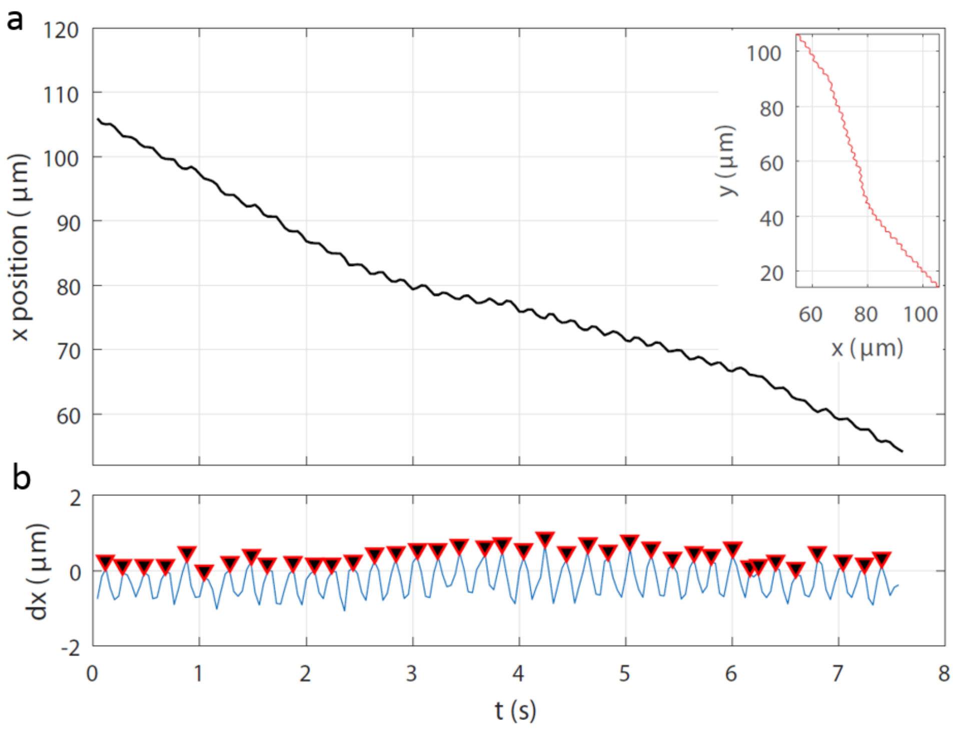

Fig. S1. Illustration of angular velocity evaluation from the 2D projection of a helical trajectory. (a) x position over time, where the inset shows the 2D projection. (b) Count number of helical turns using periodicity along dx.

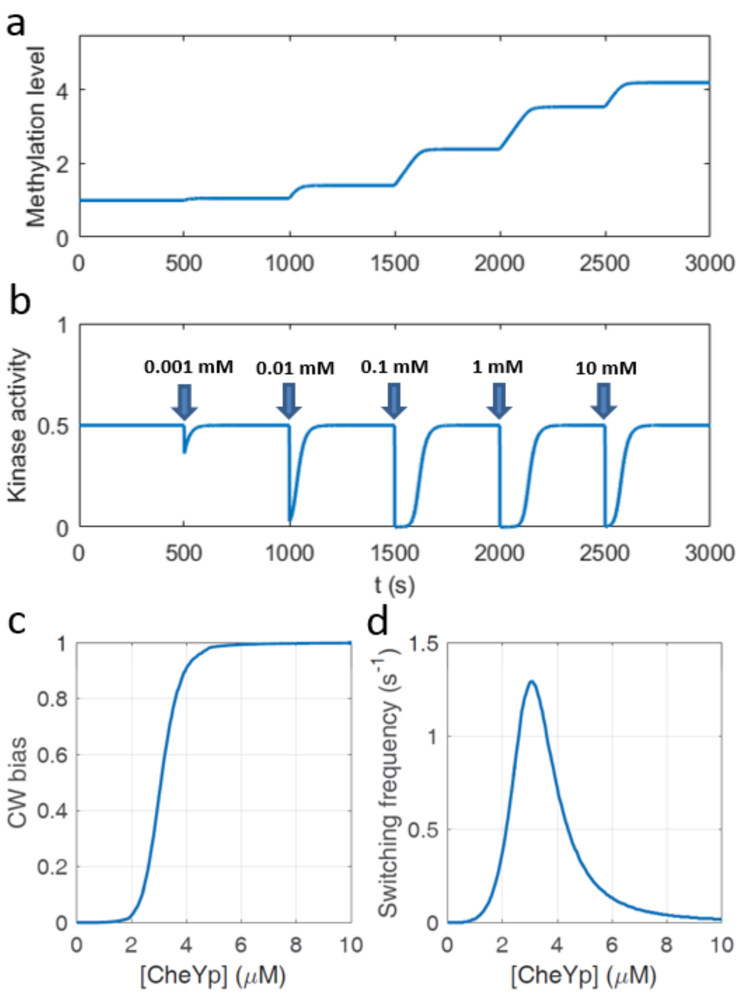

Fig. S2. Tests of bacterial chemotaxis signaling pathway model. Methylation level adaptation (a) and kinase activity dynamics (b) when applying a series of different concentration. Flagellar motor counterclockwise (CW) rotation bias (c) and rotation direction switching frequency (d) as a function of intracellular CheYp concentration.

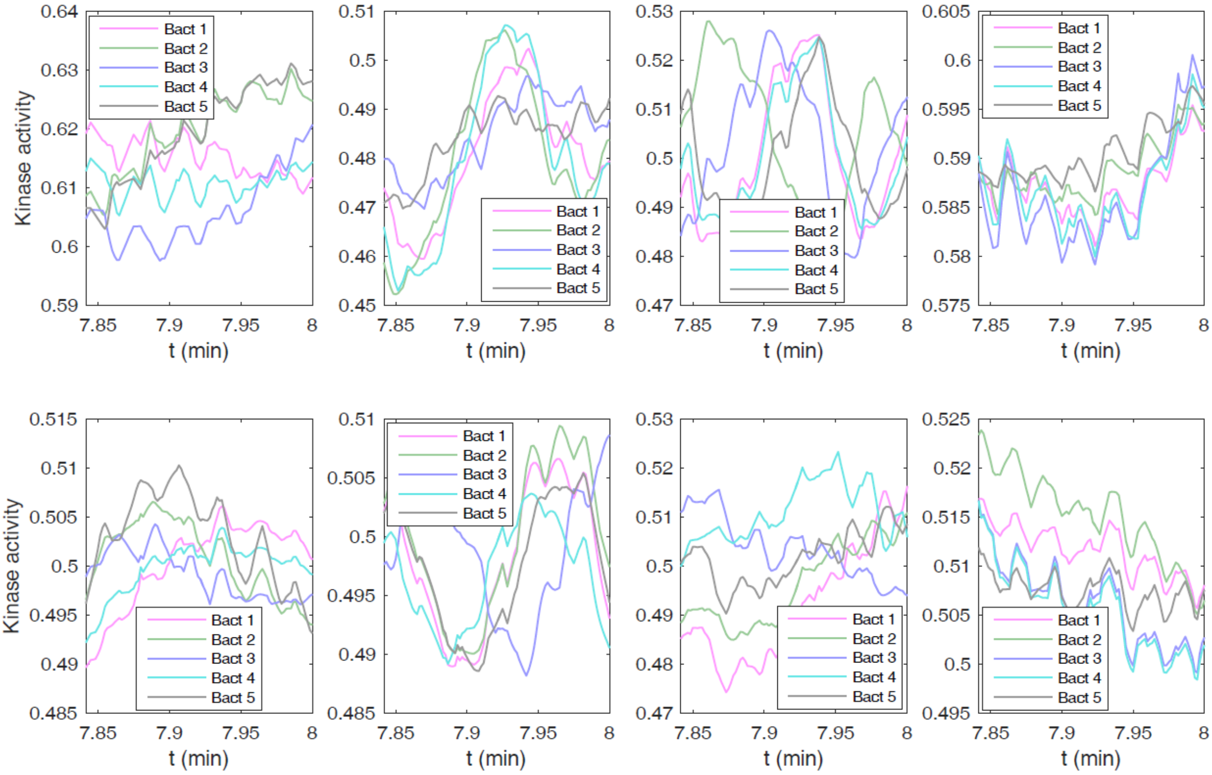

Fig. S3. Asynchronous chemotaxis signaling pathway for 10 µm diameter microswimmers (8 independent microswimmers).

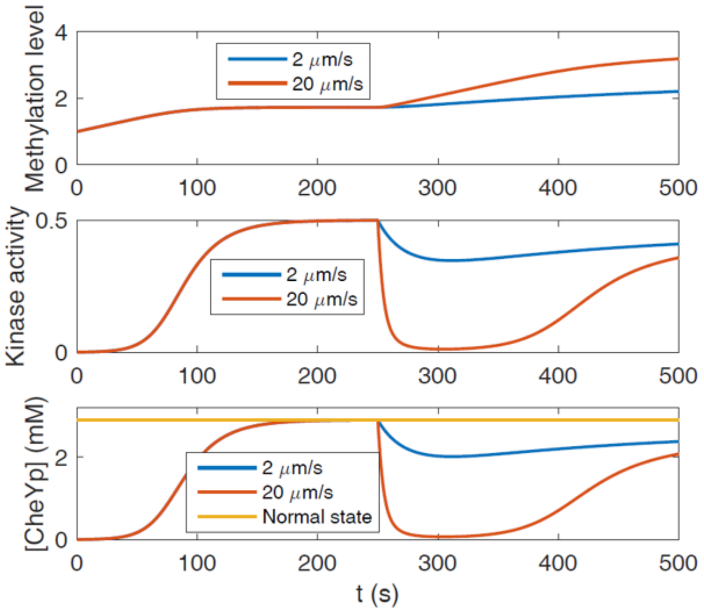

Fig. S4. Dependence of chemotaxis on swimming speed.

Supplementary Videos:

Number	Title	Description
S1	3D swimming trajectories of bacteria-driven microswimmers (experiment).	A total of 87 far-wall swimming trajectories (with time length vary from 4 to 40 seconds) were collected to characterize the 3D motion of bacteria-driven microswimmers. The separately recorded trajectories are plotted in the same space for visualization.
S2	Chemotaxis of microswimmers driven by multiple attached bacteria (simulation).	Simulated chemotaxis of microswimmers driven by multiple attached bacteria, under a linear gradient of chemoattractant along the x axis. 500 microswimmers were initialized randomly at the beginning of the simulation and the time length of the simulation is 5 minutes.
